# Effects of Ultrasonic-Assisted Extraction on the Yield and the Antioxidative Potential of *Bergenia emeiensis* Triterpenes

**DOI:** 10.3390/molecules25184159

**Published:** 2020-09-11

**Authors:** Siyuan Luo, Chen Zeng, Jiajia Li, Shiling Feng, Lijun Zhou, Tao Chen, Ming Yuan, Yan Huang, Hongyu Yang, Chunbang Ding

**Affiliations:** College of Life Science, Sichuan Agricultural University, Ya’an 625014, China; luosiyuan1998@163.com (S.L.); zc15183819422@126.com (C.Z.); Ljiajia1118@163.com (J.L.); fengshilin@outlook.com (S.F.); zhouzhou124@126.com (L.Z.); chentao293@163.com (T.C.); yuanming@sicau.edu.cn (M.Y.); shirley11hy@163.com (Y.H.); yhy4868135@163.com (H.Y.)

**Keywords:** *Bergenia emeiensis*, triterpenes, response surface method, antioxidant, CHO cells

## Abstract

This study was the first designed to evaluate the extraction and antioxidant ability of triterpenes from *Bergenia emeiensis* rhizomes. The yield of triterpenes from *B. emeiensis* was mainly affected by the concentration of ethanol, followed by the extraction time, solvent to sample ratio, and the power of ultrasound. Thus, the response surface method was applied to investigate the interaction between the two factors and to optimize the extraction process. The optimal extraction conditions were 210 W, 75% ethanol, 40 min and 25 mL/g with a maximum yield of 229.37 ± 7.16 mg UAE/g. Moreover, the antioxidant ability of triterpenes from *B. emeiensis* (TBE) was evaluated by determining the scavenging capacity on free radicals and the protection on CHO cells and *Caenorhabditis elegans* against oxidative stress. The results showed the triterpenes could clear 2,2-Diphenyl-1-picryl-hydrazyl (DPPH) radicals well and had a strong reducing power. In addition, the survival of CHO cells was higher than that of the control group as a result of reducing the reactive oxygen species (ROS) level and promoting the activities of antioxidant enzymes. In addition, TBE could also enhance the survival of *C. elegans* under H_2_O_2_ conditions. Therefore, triterpenes from *B. emeiensis* could be developed into a beneficial potential for antioxidants.

## 1. Introduction

*Bergenia emeiensis* belonged to the genus of *Bergenia*. There were more than 10 species in this genus, while *B. emeiensis* was an endemic species distributed among mountain regions alone in southwest China. Plants from *Bergenia* were usually used to treat diabetes in the rural communities of Nepal, as well as fevers and diarrhea—which might have some associations with bioactive compounds such as phenols and bergenin [1,2,3,4]. In the Emei Mountain area (Emei, China), *Bergenia emeiensis* was used as an herb to cure respiratory diseases and heal wound hemostasis. However, little research on *Bergenia emeiensis* has been reported. Plants of *Bergenia* were mainly used to extract bergenin, while the yield rate was low and other bioactive compounds were ignored. Thus, it was necessary to carry out relative experiments to analyze the pharmaceutical components. Five anthraquinones were isolated from *Bergenia hissarica* [5]. The content of arbutin was also found to vary from 17.44% to 22.59% [6]. However, there have been few reports on the triterpenes and their bioactivities.

The first and most important step to obtain active constituents from plants was extraction, which was always affected by multiple factors. In recent decades, the response surface method (RSM)—a multiple linear regression analysis method—has been widely applied to optimize the extraction process, with various advantages, such as providing clear and direct acquaintances of interactive relationship among factors. Based on the optimal extraction condition, the highest yield could be reached [7]. Meanwhile, ultrasound and microwaves were also usually used in the process to enhance the extraction rate [8,9]. Therefore, relevant technologies could be applied to assist in the extraction for some components with lower yield.

Reactive oxygen species (ROS) was a general term for single-electron reduction products of a class of oxygen in vivo, such as superoxide anions, hydroxyl radicals and hydrogen peroxide. Those free radicals could damage the cell integrity causing DNA injury, lipid peroxidation, and even aging [10,11,12]. Natural products such as polyphenols, flavonoids, polysaccharide and terpenes were proven to possess a strong clearance on free radicals in vitro and in vivo [13,14]. Hence those natural products were always developed into antioxidants in the food and medicine fields.

*B. emeiensis* as a wild efficacy resource was worth exploring its bioactive components. Therefore, in this study, the response surface method was applied to investigate the interactions of factors including ultrasonic power, concentration of ethanol, and extraction time, as well as the solvent to sample ratio. Moreover, the antioxidant ability of the triterpenes extract from *B. emeiensis* (TBE) was evaluated by its scavenging capacity on free radicals and the protection of CHO cells against H_2_O_2_. The abilities of antioxidant enzymes were also measured to elucidate the effect mechanism.

## 2. Results

### 2.1. The Effects of Four Single Factors on the Yield of Triterpenes

**Figure 1:**
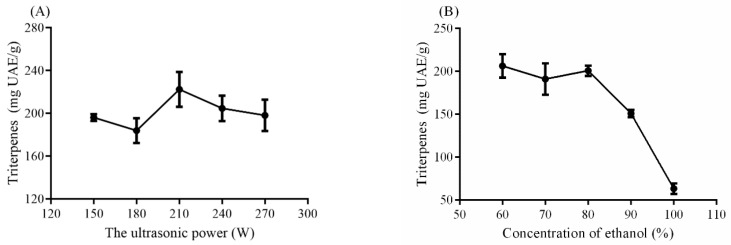

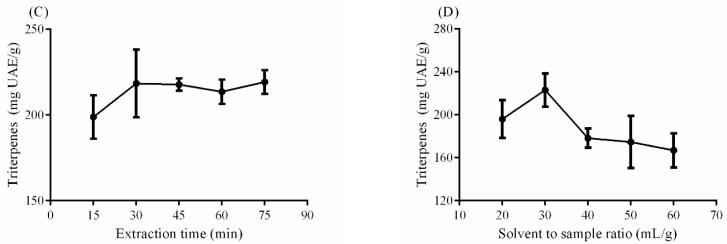
The effects of single factors on the yield of triterpenes. (**A**) ultrasonic power; (**B**) concentration of ethanol; (**C**) extraction time; (**D**) solvent to sample ratio.

Currently, ultrasonic-assisted extraction was widely used as a novel method to extract bioactive compounds from natural plants, which was easily affected by the ultrasonic power. As shown in Figure 1A, as the ultrasonic power increased, the yield of triterpenes was promoted and the highest yield was reached at the power of 210 W. The yields of 150, 180 and 270 W were significantly different from that of 210 W (*p* < 0.05). Therefore, the power of 210 W was selected as the central point of the RSM. As shown in Figure 1B, when the concentration was higher than 80%, the yield sharply decreased, and the extraction rate approached the lowest point when the ethanol was at 100%. Hence, 80% ethanol was chosen as the central point of the RSM. After prolonging the time over 80 min, the yield was decreased. Considering the energy saving, we chose 15–75 min. The central point of the extraction time was 30 min, because the yield was not changed significantly when the extraction time was over 30 min (*p* > 0.05) (Figure 1C). The solution to sample ratio also had a main effect on the extraction rate. The central point of the solution to sample ratio was obviously seen from Figure 1D. The triterpenes yield was lower than that under 30 mL/g. More solvent led to less triterpenes, which may lead to more leaching for other compounds such as polysaccharides and others. Therefore, four central points of single factors were chosen for the following RSM design.

### 2.2. Analysis on Response Surface Optimization of Triterpenes

The yield of triterpenes from *B. emeiensis* rhizomes at different experimental combinations was presented in Table 1. Four parameters—the ultrasonic power (A), concentration of ethanol (B), extraction time (C), and solvent to sample ratio (D)—were investigated. The regression model was described using the following quadratic polynomial in terms of coded values:

Y = 220.87 − 1.49A − 27.03B + 10.04C − 6.09D + 5.86AB + 2.33AC − 10.29AD + 0.96BC − 5.45BD − 8.04CD − 5.12A^2^ − 24.01B^2^ − 9.95C^2^ − 11.96D^2^ (1)

The ANOVA analysis for the fitted quadratic model is shown in Table 2. The low *p*-value (<0.0001) indicates that the regression model was highly significant. The *p*-value of lack of fit was 0.0997, which was not significant, suggesting that the model equation was suitable for predicting the yield of triterpenes from *B. emeiensis* rhizomes. R^2^ was 0.9125 and adjusted R^2^ was 0.825, which demonstrated the model had a high accuracy [15]. Furthermore, as shown in Table 2, the linear coefficients (B and C) and quadratic terms (B^2^, C^2^, D^2^) were significant (*p* < 0.05), meaning those factors had a significant effect on the yield of triterpenes. However, the *p*-values of interactions (AB, AC, AD, BC, BD and CD) were all higher than 0.05, implying that the relationship between four factors and the yield of triterpenes was not a simple linear relationship. Based on the F value, the concentration of ethanol was the main influential factor on the extraction rate, followed by extraction time, solution to sample ratio, and ultrasonic power.

**Figure 2:**
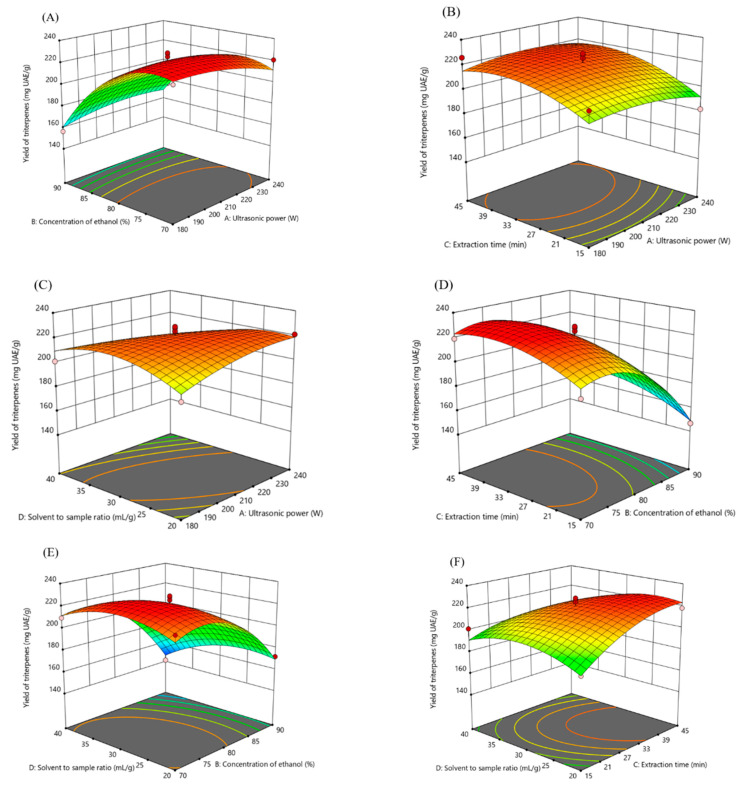
The response surface plots of the four factors interaction on the triterpenes yield (**A**) concentration of ethanol and ultrasonic power; (**B**) ultrasonic power and extraction time; (**C**) ultrasonic power and solvent to sample ratio; (**D**) extraction time and concentration of ethanol; (**E**) concentration of ethanol and solvent to sample ratio; and (**F**) extraction time and solvent to sample ratio.

Three-dimensional (3D) response surfaces were provided as an obvious graphical representation of the regression equation and the interactions among the four factors (Figure 2). The steep degree of the 3D graphs reflected the response sensitivity of the yield to the factors. A steeper 3D graph meant a larger response value [16]. The slopes in the graphs were steep, revealing how the interactions significantly influenced the yield. The increase in both ultrasonic power and concentration of ethanol (AB) accelerated the yield of triterpenes when the extraction time and solution to sample ratio were fixed (Figure 2A). As shown in Figure 2B,C, the extraction rate was not varied significantly by the interactions of AC and AD. Increasing the concentration of ethanol and prolonging a little extraction time would promote the yield of triterpenes (Figure 2D). Between the interactions of BD and CD, the solution to sample ratio played a neutral role, while extraction time had a positive effect on the yield, and slightly lowering the concentration of ethanol was also able to enhance the extraction rate (Figure 2E,F).

On the basis of RSM model, the predicted optimal extraction yield was 232.01 mg UAE/g with the conditions 217.61 W, 75.39% ethanol, 40.72 min and 25.01 mL/g (Table 1). Considering the practical operation situation, the experimental extraction condition was modified to 210 W, 75% ethanol, 40 min, and 25 mL/g. Finally, the actual yield of triterpenes in *B. emeiensis* was 229.37 ± 7.16 mg UAE/g, which was close to the predicted extraction yield, indicating that the response surface model was successfully established to optimize the extraction condition.

### 2.3. The Antioxidant Ability In Vitro

**Figure 3:**
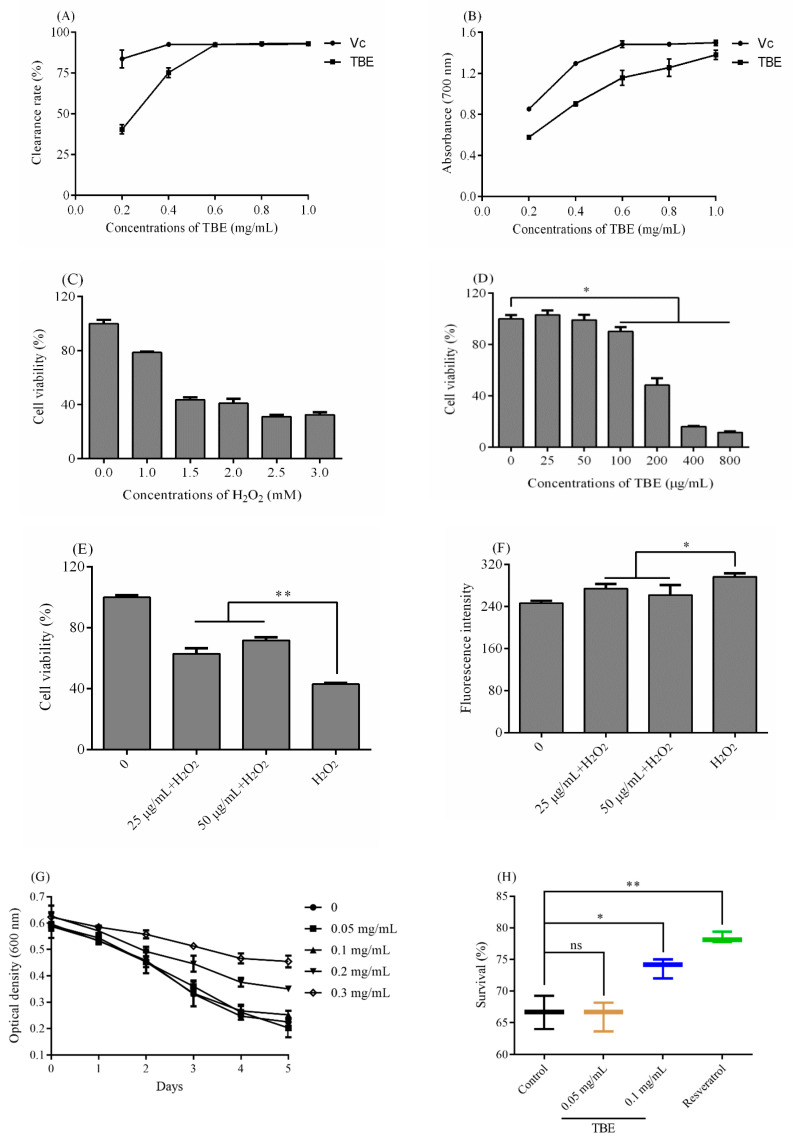
The antioxidant ability of triterpenes from *B. emeiensis* (TBE). (**A**) The clearance rate on 2,2-Diphenyl-1-picryl-hydrazyl (DPPH) radicals and (**B**) the total reducing power. (**C**) The oxidative damage model of CHO cells induced by H_2_O_2_. (**D**) The cell viability of CHO cells after treated with different concentrations TBE for 48 h. (**E**) The injury protection of TBE on CHO cells against on H_2_O_2_. (**F**) The fluorescence intensity of CHO cells after dying with 10 μM 2′7′-dichlorofluorescin diacetate (DCFH-DA). (**G**) The food clearance result and (**H**) the survival of worms under antioxidant stress. Note: * means <0.05 and ** means <0.01, and “ns” means not significant.

The total triterpenes derived from natural plants have been demonstrated to possess strong antioxidant abilities. As shown in Figure 3A,B, TBE exhibited great antioxidant power. When the concentration was higher than 0.6 mg/mL, the clearance of TBE was equivalent to the Vc with a maximum scavenging rate of 93% (Figure 3A). A high value of absorbance at 700 nm represented a strong total reducing power. The value of ascorbic acid (Vc) was higher than that of TBE, but the total reducing power of TBE was boosted with an increased concentration, while the absorbance of Vc was becoming flat (Figure 3B). Hence, TBE showed an excellent antioxidant capacity, and could be a potential antioxidant.

### 2.4. The Protection on CHO against H_2_O_2_

H_2_O_2_ was a trigger for inner oxidative injury, as it could induce the formation of reactive oxygen species (ROS) [17]. Recently, reports have proven that bioactive compounds from natural plants could scavenge free radicals well, then lower the level of ROS to protect organs [18]. As shown in Figure 3D, under the H_2_O_2_ concentration of 1.5 mM, the survival of CHO cells was close to 50%. Hence, this concentration was suitable for the following experiments. When the concentration was higher than 100 μg/mL, TBE showed an acute cell cytotoxicity on CHO cells, while 25 and 50 μg/mL had no significant effect on the growth of CHO cells. Therefore, 25 and 50 μg/mL were used to detect the protection of TBE. The viability of CHO cells was low when cells were under the condition of H_2_O_2_ (Figure 3E). However, the cell viability was promoted when the cells were pretreated with TBE for 48 h, which showed a significance compared to the H_2_O_2_ group (*p* < 0.01). Thus, TBE showed a great protection on CHO cells against oxidative injury.

### 2.5. TBE Could Reduce ROS Level and Promote the Antioxidant Ability

A high level of ROS would induce cell injury, lipid peroxidation and even chromosome variation [19]. After being treated with TBE for 48 h, the level of ROS descended compared to the H_2_O_2_ group, although the fluorescence intensity of both groups was higher than that of the control group (Figure 3F). To evaluate the antioxidant system of CHO cells, the activities of two antioxidant enzymes and the content of Malondialdehyde (MDA), as well as the total antioxidant capacity (T-AOC), were assessed. The T-AOC of the administration group was higher than that of the control and H_2_O_2_ groups, which meant that TBE could enhance the whole antioxidant capacity in CHO cells (Table 3). The activities of two main antioxidant enzymes catalase (CAT) and superoxide dismutase (SOD) in TBE group were extraordinarily strange compared to other groups (Table 3). Hence, TBE could strengthen the activities of antioxidant enzymes, effectively clearing the free radicals induced by H_2_O_2_, and lowering the level of ROS as well as MDA. Therefore, cells in the TBE group suffered less compared to those of the H_2_O_2_ group, demonstrating TBE might enhance the antioxidant system to activate the antioxidant enzymes (Table 3).

### 2.6. TBE Could Enhance the Survival of C. elegans under Antioxidant Stress

Since TBE showed a strong antioxidant ability in vitro and protection on CHO against H_2_O_2_, the body in vivo environment was complex. *C. elegans* was widely used as a model organ to screen natural antioxidants [20]. As shown in Figure 3G, 0.2 and 0.3 mg/mL TBE negatively affected the food clearance, while 0.05 and 0.1 mg/mL showed no obvious effect. Hence, 0.05 and 0.1 mg/mL were chosen as the suitable concentrations for the oxidative stress assay. When the worms were exposed to H_2_O_2_, the survival of control group was 66.64 ± 2.62%, which was not significantly different from the survival rate of worms treated with 0.05 mg/mL. Nevertheless, the worms pretreated with 0.1 mg/mL of TBE could well enhance the survival to 73.73 ± 1.55% (Figure 3H). Therefore, TBE also protected *C. elegans* against H_2_O_2_, thus exhibiting great antioxidant activity in vivo.

## 3. Materials and Methods

### 3.1. Materials and Chemicals

*B. emeiensis* was collected from Mount Emei, Sichuan, China, at an altitude of 1300–1500 m. The herbarium was collected and stored in the specimen room of the College of Life Science, Sichuan Agricultural University. The rhizomes were washed by up-water three times, then dried at 50 °C before being ground into fine powder sieving 60 meshes.

2,2-Diphenyl-1-picryl-hydrazyl (DPPH) was purchased from Sigma Chemical Co. (St. Louis, MO, USA). Ethanol, acetic acid, vanillin, ferric chloride, and perchloric acid were purchased from the Chengdu Kelong Chemical Factory (Chengdu, China). The Reactive Oxygen Species Assay Kit (ROS) was purchased from Beyotime Biotechnology (Shanghai, China). The total antioxidant capacity assay kit (T-AOC) and the total protein assay kit (with standard: BCA method) were bought from Nanjing Jiancheng Bioengineering Institute (Nanjing, China). Fetal bovine serum (FBS) was obtained from Gibco. Dulbecco’s modified eagle medium (DMEM), PBS, digestive enzyme and Penicillin-Streptomycin solution (10×) were purchased from Hyclone (Logan, UT, USA). All chemicals were analytical grade.

### 3.2. Determination of Triterpenes

The triterpenes content from *B. emeiensis* was detected by perchloric acid-vanillin colorimetry [21]. Briefly, 0.05 mL sample solution was mixed with 0.1 mL vanillin-acetic acid (2.5%) and 0.2 mL perchloric acid, then placed at 60 °C. After 15 min incubation, acetic acid was added into the solution to 1 mL standing for 10 min. Finally, the absorbance of the mixture was read at 550 nm by a microplate reader (Spectramax M2, San Francisco, CA, USA). The total triterpenes were calculated in accordance with a standard curve, using ursolic acid as the reference. The content of triterpenes was expressed as milligrams of ursolic acid equivalents per gram (mg UAE/g) of dry sample.

### 3.3. Ultrasound-Assisted Extraction (UAE)

The triterpenes were extracted by ethanol with the assistance of ultrasound. The temperature of the ultrasound was fixed at 45 °C. Four factors were observed. Different concentrations of ethanol (60–100%) were mixed with fine powder of *B. emeiensis*, making the solution to sample ration range from 1:20 to 1:60 g/mL. The mixture was placed at variable ultrasonic power (150–210 W) for different times in the range 15–75 min. After extraction, the triterpenes content in the sample solution was determined according to the method of 3.2. Based on the results, three suitable extraction conditions of four factors were chosen for the following response surface methodology design. All measurements were performed for four replications.

### 3.4. Response Surface Methodology (RSM) Design

The optimization experiments for UAE of triterpenes from *B. emeiensis* were performed by employing a three-level-four-factor Box-Behnken design (BBD) with RSM. The extraction yield of triterpenes was taken as the response. The coded and actual levels of the four factors were presented in Table 1. The mean values of dependent parameters obtained from the four-replication measurements were fitted to a second order polynomial model as follows:

(2) $$Y=\beta_{0}+\sum_{i=1}^{4}\beta_{i}X_{i}+\sum_{i=4}^{4}\beta_{ii}X_{i}^{2}+\sum_{i<j=2}^{4}\beta_{ij}X_{i}X_{j}$$

where *Y* was the predicted extraction yield; *β*_0_ denoted a constant; *β_i_*, *β_ii_*, and *β_ij_* denoted coefficients of the linear, quadratic, and interaction terms, respectively; and *X_i_* and *X_j_* denoted independent variables. An analysis of variance (ANOVA) was performed to detect the individual linear, quadratic and interaction regression coefficients (*β*) using Design Expert 11 trial version (Stat-Ease Inc., Minneapolis, MN, USA). The significance of the dependent variables was statistically analyzed by computing *p* value.

### 3.5. Antioxidant Activity Assays In Vitro

The triterpenes were extracted at the optimal condition calculated according to the RSM results. The extraction solution was filtered using a Buchner funnel, then the filtrate was dried in a vacuum freeze drier. The triterpenes from *B. emeiensis* (TBE) were placed at −20 °C for the following experiments.

#### 3.5.1. DPPH Radicals Scavenging

The DPPH free radicals scavenging ability was determined according to the previous method [22] with some slight modifications. Briefly, TBE was resolved in ethanol to different concentrations (0–1.0 mg/mL). An amount of 30 μL TBE sample solution was mixed with 170 μL of DPPH ethanol solution (0.8 mM), and ascorbic acid (Vc) was used as the positive control. The mixture solutions were placed in the dark at room temperature for 10 min, before the absorbance was read at 517 nm. The DPPH radical scavenging ability was calculated by the following formula:

DPPH radical scavenging activity (%) = (1 − A_1_/A_0_) × 100 (3)

where A_1_ was the absorbance of the mixture solutions with different concentrations of TBE, and A_0_ was the control group and replaced sample solution with distilled water.

#### 3.5.2. Reducing Power

The reducing power was associated with the antioxidant ability to some degree. Therefore, the previous description was conducted to determine the reducing power of TBE, with some modifications [23]. First, 0.2 mL of the TBE sample solution (Vc was used as the positive control group) was mixed with 0.5 mL K_3_[Fe(CN_6_)] (1% *w*/*v*) and 0.5 mL phosphate buffer (0.2 M), then placed at 50 °C for 20 min. Subsequently, the reaction solution was cooled at 0 °C for 5 min before adding 0.5 mL trichloroacetic acid (10% *w*/*v*). Then, a 0.5 mL aliquot of supernatant fluid was mixed with 0.1 mL FeCl_3_ (0.1% *w*/*v*) and 0.5 mL distilled water. The absorbance of the mixture was read at 700 nm by a microplate reader. The results were expressed as the actual absorbance. An increasing absorbance indicated an ascending reducing power.

#### 3.5.3. Cell Culture

Chinese hamster ovary cells (CHO cells) were obtained from the Biotechnology Center of Sichuan University (Sichuan, China). CHO cells were cultured in DMEM containing 10% FBS and 1% penicillin-streptomycin (10×) at 37 °C in a 5% CO_2_ atmosphere. When the cells were in the logarithm growth period, the cells were digested from the culture flask and washed with PBS twice before being plated into microplates for the following experiments.

#### 3.5.4. Establishment of an Oxidative Damage Model

H_2_O_2_ was usually used as a trigger for oxidative damage [19]. CHO cells were adjusted to the density of 1 × 10^5^ cells/mL, and 90 µL cell suspension was added into 96 microplates with the edge filled with 100 µL PBS. After 12 h, different 10 µL concentrations of H_2_O_2_ were added. The plates were incubated at 37 °C for 5 h, before 100 µL fresh medium was used to totally replace the old solution. Then, 10 µL cell counting kit-8 (CCK-8) solution was added into the wells and incubated for 30 min before the absorbance of each well was read at 450 nm. The CCK-8 was purchased from Wuhan Boster Biological Technology., LTD (Wuhan, China). Cell viability was measured according to the following equation:

Cell viability (%) = A_1_/A_0_ × 100 (4)

where A_1_ was the absorbance of the cell well with cells, CCK-8 solution, and H_2_O_2_; and A_0_ was the absorbance of a well with cells, CCK-8 solution, and PBS replaced with H_2_O_2_.

#### 3.5.5. The Cytotoxicity of TBE

The CCK-8 method was performed to detect the cell cytotoxicity of TBE on CHO cells. TBE was resolved in DMSO and the final concentration of DMSO was lower than 0.05%. CHO cells were adjusted to the destiny of 1 × 10^5^ cells/mL. Then, 90 µL cell suspension was plated into wells with the edge filled with PBS. After incubation for 12 h, 10 µL sample solution and 0.05% DMSO was added into the wells for 48 h as the negative control. Then, the old solution was totally replaced with fresh medium containing 10% CCK-8. After 30 min, the absorbance of each well was read at 450 nm. The cell viability was calculated according to the following formula:

Cell viability (%) = A_1_/A_0_ × 100 (5)

where A_1_ was the absorbance of the cell well with cells, CCK-8 solution, and sample solution; and A_0_ was the absorbance of a well with cells, CCK-8 solution, and PBS replaced with sample solution.

#### 3.5.6. The Injury Protection of TBE on CHO Cells against H_2_O_2_

CHO cells were plated to microplates at a density of 1 × 10^4^ cells per well. A different concentration of TBE was added to each well for 48 h. Subsequently, the solution was removed before adding fresh medium and H_2_O_2_ for 5 h. All solutions were totally replaced with new media and CCK-8 assays. After 30 min, the absorbance of the wells was read at 450 nm, and the cell viability was determined using the following equation:

Cell viability (%) = A_1_/A_0_ × 100 (6)

where A_1_ was the absorbance of the cell well with cells, CCK-8 solution, and sample solution; and A_0_ was the absorbance of a well with cells, CCK-8 solution, and PBS replaced with sample solution.

#### 3.5.7. Determination of ROS Level

CHO cells were suspended with high-glucose DMEM to the concentration of 1 × 10^6^ cells/mL. Then, 1.8 mL of cell suspension was added into 6 microplates. After 12 h of incubation, 0.2 mL TBE and DMSO solution (0.05%, *v*/*v*) were added into each well for 48 h, respectively. Then, the solution was totally removed and H_2_O_2_ was added into each well for 5 h. The cells were washed with PBS twice to remove H_2_O_2_. Subsequently, ROS assay was added for 45 min. Then, the cells were washed with PBS twice to remove the assay. The fluorescence intensity of each well was read through a microplate reader.

#### 3.5.8. The Measurement of T-AOC and Antioxidant Enzymes as well as the Content of MDA

The CHO cells were adjusted to the concentration of 1 × 10^6^ cells/mL. An amount of 1.8 mL cell suspension was added into 6-well microplates for 12 h of incubation. Then, 0.2 mL of TBE and DMSO solution (0.05%, *v*/*v*) as blank control were added into each well, respectively. After 48 h, the solution was totally removed, and H_2_O_2_ was added into each well for 5 h. Then, digestive enzyme was added to digest the cells from the flask, and the cells were washed twice with PBS. The cells were disrupted by an ultrasonic homogenizer (Scientz-IID, Ningbo, China). Then, the protein content of the supernatant was determined according to the BCA kit. T-AOC and antioxidant enzymes, as well as the content of MDA, were evaluated in accordance with the kits’ instructions (Nanjing Jiancheng Bioengineering Institute, Nanjing, China).

### 3.6. The Antioxidant Assays In Vivo

#### 3.6.1. The Maintenance of *Caenorhabditis elegans*

Wild-type N_2_ *Caenorhabditis elegans* (*C. elegans*) and *Escherichia coli* OP50 strain were kindly provided from the *Caenorhabditis* Genetics Center (CGC). *C. elegans* were maintained on nematode growth medium (NGM) agar plates, fed with *E. coli* OP50 as a food source, and maintained at 20 °C. Synchronized *C. elegans* were obtained using the sodium hypochlorite method [24].

#### 3.6.2. Food Clearance Assay

A food clearance assay was carried out to find the no-toxic concentrations of TBE on *C. elegans* [21] Synchronized worms were added into liquid medium with *E. coli* OP50 and 5-fluorouracil (the final concentration was 25 μm) and the optical density was adjusted to 0.6–0.7. Then, TBE was transferred into each well. The plate was placed at 20 °C for 5 days. The OD of the plate was read at 600 nm every day.

#### 3.6.3. Oxidative Stress Assay

TBE was mixed with OP50 and then spread on the NGM plate. Pregnant nematodes were placed on the plate for 2 h to lay eggs. Resveratrol (100 μg/mL) was used as the positive control. Then, the L1 worms were transferred to the NGM containing TBE for 48 h. Subsequently, the worms were exposed to H_2_O_2_ (0.5% *v*/*v*) for 3 h. The live worms were counted.

### 3.7. Statistical Analyses

The data obtained in this study were analyzed statistically using Student’s *t*-test (*t*-test) and ANOVA (GraphPad Prism 6) (GraphPad Software, Inc., La Jolla, CA, USA). Design-Expert 11 software (trial version, State-Ease Inc., Minneapolis, MN, USA) was used to analyze the experimental results of the response surface design. All experimental results were expressed as mean±standard deviation (SD), *n* = 4. A difference at the *p* < 0.05 level was considered to be significantly different, and *p* < 0.01 was very significant.

## 4. Discussion

Various factors affected the extraction rate of the bioactive compounds from natural plants. As for the extraction of triterpenes, different polar solvents were used, such as ethanol and methanol, among which ethanol was widely used for its cheapness and low toxicity. Vaghela and Pandita used ethanol to obtain the highest yield of triterpenes (59.86 ± 0.005%) from *Gymnema sylvestre* [25]. In this study, ethanol (lower than 80%) promoted the extraction rate of TBE, while a higher concentration reduced the yield. Moreover, a suitable solvent to sample ratio also helped in extracting the compounds. Usually, the appropriate ratio for extracting bio-compounds from plants ranged from 10 to 60 mL/g, and the ratio between 20 and 40 was also selected as a suitable ratio in this study. With the assistance of ultrasound, the yield was promoted. At a power of 210 W, the extraction rate of TBE was the highest, indicating that the ultrasonic-assisted extraction method could enhance the leaching rate of triterpenes and have a similar effect on other active compounds [26]. A suitable prolonged extraction time could also enhance the yield of TBE. Considering that the four factors showed a different effect on the yield, the response surface method was used to investigate the interactions among the factors, because RSM had been successfully applied to elucidate the interactions and optimize the process for other compounds in many plants [27]. Under the optimal extraction conditions obtained from RSM, the gained rate of TBE was 229.37 ± 7.16 mg UAE/g to the maximization. Hence, RSM was still the most effective method to optimize the extraction process from natural plants.

Natural triterpenes have been proven to be an excellent antioxidant. Triterpenes from the aerial parts of *Salvia barrelieri* could clear DDPH radicals [28]. TBE also showed a good scavenging ability on DPPH and had a strong reducing power. Although the antioxidant ability in vitro was great, the effect on cell level still needed further investigation to prove this. H_2_O_2_ was the trigger of ROS, inducing the formation of harmful radicals such as •OH and O_2_^−^, causing cell damage, chromosome variation, and even cancer [29]. Antioxidants could scavenge radicals and lower the level of ROS. On the other hand, some antioxidants could stimulate the antioxidant systems to defend the increasing free radicals [30]. In this study, pretreated with TBE, the cell viability was enhanced compared with the injury group. Further experiments showed that the abilities of antioxidant enzymes such as CAT and SOD were promoted—both of which cleared the free radicals inside the cells and reduced the damage caused by CHO. Thus, the total antioxidant capacity (T-AOC) of the CHO cells was improved and the ROS level was reduced for the increasing abilities of antioxidant enzymes. Smina et al. found that the triterpenes from *Ganoderma lucidum* could scavenge free radicals well in vitro and exhibit a good protective effect in splenic lymphocytes by promoting the activity of antioxidant enzymes and lowering ROS levels [23]. When the organ was exposed to H_2_O_2_, the ROS sharply increased, damaging the cells and even causing death. TBE could protect *C. elegans* against H_2_O_2_ and increase the survival rate, like other natural antioxidants [31]. Hence, TBE was assumed to exert strong antioxidant ability by lifting the abilities of antioxidant enzymes then lowering the level of increasing ROS. However, whether TBE could directly clear the increasing free radicals inside the cells still needed further experiments to investigate the links, and the inner antioxidant capacity still needed more investigations to explore the mechanism.

## 5. Conclusions

The response surface method was successfully applied to investigate the interactions and optimize the extraction process. The concentration of ethanol played a main role on the extraction rate. Under the optimal conditions—210 W, 75% ethanol, 40 min, and 25 mL/g—the yield of triterpenes from *B. emeiensis* was 229.37 ± 7.16 mg UAE/g. TBE could efficiently clear DPPH radicals in vitro with a maximum scavenging rate of 93%. Moreover, compared with the injury group, TBE could protect CHO cells from the damage of H_2_O_2_ and promote their survival. The levels of ROS and MDA in CHO cells were reduced after being treated with TBE for 48 h. Further results demonstrated that TBE could simulate the activities of antioxidant enzymes so as to clear the increasing free radicals. TBE also prolonged the survival rate from 66.64 ± 2.62% to 73.73 ± 1.55% under H_2_O_2_ stress. Therefore, the triterpenes from *B. emeiensis* were proven to possess a strong antioxidant ability; thus, they could have potential in the field of food as well as in the medicine industry.

## Figures and Tables

**Table 1:** The response surface method (RSM) design and results.

Run	A-Ultrasonic Power (W)	B-Concentration of Ethanol (%)	C-Extraction Time (min)	D-Solvent to Sample Ratio (mL/g)	Yield of Triterpenes (mg UAE/g)
1	180	70	30	30	223.73
2	210	80	30	30	227.19
3	210	90	30	20	171.88
4	180	80	15	30	208.80
5	210	80	15	40	201.34
6	240	70	30	30	220.68
7	240	80	30	20	220.98
8	240	90	30	30	176.82
9	240	80	45	30	208.13
10	210	90	15	30	146.69
11	210	80	15	20	186.76
12	240	80	15	30	181.84
13	180	80	30	20	195.72
14	210	70	15	30	197.70
15	210	80	30	30	200.20
16	240	80	30	40	185.27
17	210	90	30	40	141.10
18	210	70	30	40	209.50
19	180	80	30	40	201.15
20	210	70	30	20	218.47
21	210	80	30	30	224.45
22	210	70	45	30	219.19
23	210	80	30	30	224.44
24	210	80	45	20	218.04
25	180	80	45	30	225.76
26	180	90	30	30	156.42
27	210	90	45	30	172.03
28	210	80	30	30	228.06
29	210	80	45	40	200.44
pred	217.61	75.39	40.72	25.01	232.01
exp	210	75	40	25	229.37 ± 7.16

**Table 2:** ANOVA for response surface quadratic model.

Source	Sum of Squares	Mean Square	F Value	*p*-Value
Model	15651.6	1117.97	10.43	<0.0001 ***
A	26.62	26.62	0.25	0.626
B	8765.46	8765.46	81.74	<0.0001 ***
C	1209.16	1209.16	11.28	0.0047 **
D	444.57	444.57	4.15	0.0611
AB	137.38	137.38	1.28	0.2767
AC	21.76	21.76	0.2	0.6592
AD	423.28	423.28	3.95	0.0669
BC	3.7	3.7	0.035	0.8553
BD	118.88	118.88	1.11	0.3102
CD	258.84	258.84	2.41	0.1426
A^2^	170.29	170.29	1.59	0.2282
B^2^	3739.29	3739.29	34.87	<0.0001 ***
C^2^	641.6	641.6	5.98	0.0283 *
D^2^	927.26	927.26	8.65	0.0107 *
Residual	1501.22	107.23		
Lack of fit	956.54	95.65	0.7	0.7044
Pure error	544.67	136.17		
Cor total	17152.82			

Note: * means *p* < 0.05; ** means <0.01 and *** means *p* < 0.001.

**Table 3:** The activities of antioxidant enzymes and content of Malondialdehyde (MDA) as well as total antioxidant capacity.

	0	25 μg/mL + H_2_O_2_	50 μg/mL + H_2_O_2_	H_2_O_2_
CAT (U/mgprot)	41.11 ± 1.72	68.55 ± 5.87 ***	76.47 ± 10.01 ***	47.43 ± 2.35
SOD (U/mgprot)	86.77 ± 5.18	160.88 ± 4.23 ***	157.89 ± 7.16 ***	132.33 ± 6.05
T-AOC (U/mgprot)	4.57 ± 0.84	7.40 ± 1.46 **	9.81 ± 0.81 ***	3.11 ± 1.89
MDA (nmol/mgprot)	145.69 ± 16.39	237.02 ± 15.52 ***	213.44 ± 19.43 ***	300.88 ± 16.60

Note: ** means *p* < 0.01 and *** means *p* < 0.001 compared with the group of H_2_O_2_.
